# Performance of the BinaxNOW coronavirus disease 2019 (COVID-19) Antigen Card test relative to the severe acute respiratory coronavirus virus 2 (SARS-CoV-2) real-time reverse transcriptase polymerase chain reaction (rRT-PCR) assay among symptomatic and asymptomatic healthcare employees

**DOI:** 10.1017/ice.2021.20

**Published:** 2021-01-25

**Authors:** Allison E. James, Trent Gulley, Atul Kothari, Kasey Holder, Kelley Garner, Naveen Patil

**Affiliations:** 1 Epidemic Intelligence Service, Centers for Disease Control and Prevention, Atlanta, Georgia; 2 Arkansas Department of Health, Little Rock, Arkansas; 3 St Bernards Medical Center, Jonesboro, Arkansas

## Abstract

The sensitivity of the BinaxNOW coronavirus disease 2019 (COVID-19) Ag Card test (BinaxNOW) was 51.6% among asymptomatic healthcare employees relative to real-time reverse transcriptase polymerase chain reaction (rRT-PCR). The odds of a positive BinaxNOW test decreased as cycle threshold value increased. BinaxNOW could facilitate rapid detection and isolation of asymptomatically infected persons in some settings while rRT-PCR results are pending.

On October 1, 2020, the Arkansas Department of Health (ADH) was notified of 159 cases of coronavirus disease 2019 (COVID-19) diagnosed in the preceding 2 weeks among employees of an acute-care hospital with 438 beds (hospital X). These employees were tested for severe acute respiratory coronavirus virus 2 (SARS-CoV-2) if they had symptoms or contact with a person who tested positive. During September 17–30, 2020, the county where hospital X is located reported a real-time reverse transcriptase polymerase chain reaction (rRT-PCR)test positivity rate of 11% (Centers for Medicare & Medicaid Services data). An ADH team was deployed to hospital X on October 2 to assist with SARS-CoV-2 testing of hospital employees and to advise on infection prevention and control measures. As part of the testing strategy, duplicate nasal swab specimens were collected from all clinical staff and volunteer nonclinical staff for concurrent testing with the BinaxNOW COVID-19 Ag Card tests (BinaxNOW) and the PerkinElmer SARS-CoV-2 real-time RT-PCR assay (rRT-PCR) (PerkinElmer, Waltham, MA). Employees who tested positive with BinaxNOW were immediately isolated and excluded from work while rRT-PCR results were pending. BinaxNOW test sensitivity, specificity, positive and negative predictive values, and concordance were calculated relative to rRT-PCR results for symptomatic and asymptomatic participants to determine the utility of BinaxNOW for detecting asymptomatic SARS-CoV-2 positive persons in an outbreak setting.

BinaxNOW is a rapid point-of-care lateral flow immunoassay that detects SARS-CoV-2 nucleocapsid protein antigen. The test was granted emergency use authorization (EUA) by the US Food and Drug Administration (FDA) on August 26, 2020, with intended use in persons with suspected COVID-19 within 7 days of symptom onset.^1^ According to product information, the test achieves 97.1% sensitivity and 98.5% specificity when used within 7 days of symptom onset.^2^ However, a statement issued by the FDA indicated that the test can be used off-label in asymptomatic persons if highly sensitive tests (eg, rRT-PCR tests) are not feasible or if turn-around times are prolonged.^2^

We report the diagnostic test characteristics of the BinaxNOW test relative to the PerkinElmer SARS-CoV-2 real-time RT-PCR test during a COVID-19 outbreak.

## Methods

### Employee testing procedure

During October 2–9, 2020, all employees providing patient care, except those who had tested positive for SARS-CoV-2 within the previous 90 days, were required by their employer to participate in widespread dual testing (BinaxNOW and rRT-PCR tests); testing for non-clinical staff was optional. Among ∼3,300 total employees (clinical and non-clinical), 2,339 participated in dual SARS-CoV-2 testing during the event. Employees who were tested also provided self-documented symptoms (ie, fever, cough, sore throat, dyspnea, chills, headache, muscle aches, vomiting, abdominal pain, diarrhea, or loss of taste or smell) and onset date of any symptom on laboratory reporting forms. Employees received testing at dedicated stations within the hospital through mobile teams deployed to hospital units or drive-through parking lot stations.

### Laboratory methods

Two nasal swab samples were collected in random order from participating employees by trained hospital staff. Specimens for each test were collected by rotating the same swab at least 5 times, and for 15 seconds, inside both nares. One swab was provided for use in the BinaxNOW COVID-19 Ag Card test kit (Abbott Diagnostics, Scarborough, ME); the other was a flocked specimen collection swab (iClean) used for rRT-PCR testing. The latter sample was placed in viral transport media or sterile saline, transported on ice to the ADH Public Health Laboratory, and tested using the PerkinElmer SARS-CoV-2 real-time RT-PCR assay. Cycle threshold (Ct) values were obtained for 2 viral gene targets (*N* and *Orf1*); Ct values below 42 cycles for either *N* or *Orf1* were considered positive for SARS-CoV-2. The second nasal sample was immediately placed into a BinaxNOW test card and run per label instructions by trained laboratory employees of hospital X. Hospital X reported antigen test results to the ADH. All paired samples were successfully tested.

### Statistical analyses

Test result data were combined into a single data set and stored on an ADH secure server. The diagnostic test parameters of the BinaxNOW test were calculated relative to the PerkinElmer rRT-PCR test, utilized as the reference standard, for all employees, symptomatic employees, and asymptomatic employees. Univariate logistic regression analyses were conducted to compare Ct values obtained from positive rRT-PCR tests with BinaxNOW test results. Ct values for N and Orf1 approximated one another (Pearson correlation coefficient, 0.99); therefore, only values for the N target are reported. Ct analyses were completed with SAS version 9.4 software (SAS Institute, Cary, NC).

### Ethics

The University of Arkansas for Medical Sciences Institutional Review Board granted an exemption from review for this study. The ADH Science Advisory Committee further reviewed and approved of this activity. Additionally, this activity was reviewed by Centers for Disease Control and Prevention (CDC) and was conducted consistent with applicable federal law and CDC policy (see eg, 45 C.F.R. part 46, 21 C.F.R. part 56; 42 U.S.C. §241(d); 5 U.S.C. §552a; 44 U.S.C. §3501 et seq).

## Results

During the testing event, 2,339 employees underwent paired PerkinElmer rRT-PCR and BinaxNOW testing. Employees participating in testing were aged 16–89 years (median, 37 years). The day of sample collection, 2,224 (95.1%) persons were asymptomatic and 115 (4.9%) reported at least 1 symptom. Overall, 152 (6.5%) employees tested positive with rRT-PCR; rRT-PCR test positivity among symptomatic and asymptomatic employees was 20.9% (n = 24) and 5.8% (n = 128), respectively (Table 1). Overall, among the symptomatic employees, 94 (81.7%) were experiencing only 1 symptom and 21 (18.3%) reported 2–6 symptoms (Supplementary Table S1 online).

**Table 1.** Comparison of Test Results From All Hospital X Employees, Symptomatic Employees, and Asymptomatic Employees at the Time of Sample Collection

Variable	Overall			Symptomatic^a^			Asymptomatic		
	rRT-PCR^b^			rRT-PCR			rRT-PCR		
	Positive	Negative	Row Total (%)	Positive	Negative	Row Total (%)	Positive	Negative	Row Total (%)
Antigen^c^ positive	86	3	89 (3.8)	20	0	20 (17.4)	66	3	69 (3.1)
Antigen negative	66	2,184	2,250 (96.2)	4	91	95 (82.6)	62	2,093	2,155 (96.9)
Column total (%)	152 (6.5)	2,187 (93.5)	2,339 (100)	24 (20.9)	91 (79.1)	115 (100)	128 (5.8)	2,096 (94.2)	2,224 (100)

a A person was considered symptomatic if they had at least 1 of the following: fever, cough, sore throat, dyspnea, chills, headache, muscle aches, vomiting, abdominal pain, diarrhea, loss of taste, or loss of smell.

b PerkinElmer SARS-CoV-2 rRT-PCR diagnostic assay.

c BinaxNOW COVID-19 Ag Card test.

The BinaxNOW test sensitivity relative to the rRT-PCR test was 56.6% overall but was 83.3% in symptomatic persons and 51.6% in asymptomatic persons (Table 2). Specificity was high (≥99%) among both symptomatic and asymptomatic employees. Concordant results were obtained in 2,270 (97.1%) employees: 111 symptomatic (96.5%) and 2,159 asymptomatic (97.1%).

**Table 2.** Test Parameters of the BinaxNOW Card Test Relative to the PerkinElmer rRT-PCR Assay

Variable	Overall	Symptomatic	Asymptomatic
Sensitivity, %	56.6 (48.7–64.5)^a^	83.3 (68.4–98.2)	51.6 (42.9–60.2)
Specificity, %	99.9 (99.7–100)	100 (100–100)	99.9 (99.7–100)
Positive predictive value, %	96.6 (92.9–100)	100 (100–100)	95.7 (90.8–100)
Negative predictive value, %	97.1 (96.4–97.8)	95.8 (91.8–99.8)	97.1 (96.4–97.8)
Concordance, %	97.1 (96.4–97.7)	96.5 (93.2–99.9)	97.1 (96.4–97.8)

a 95% confidence intervals shown in parentheses.

**Fig 1.**
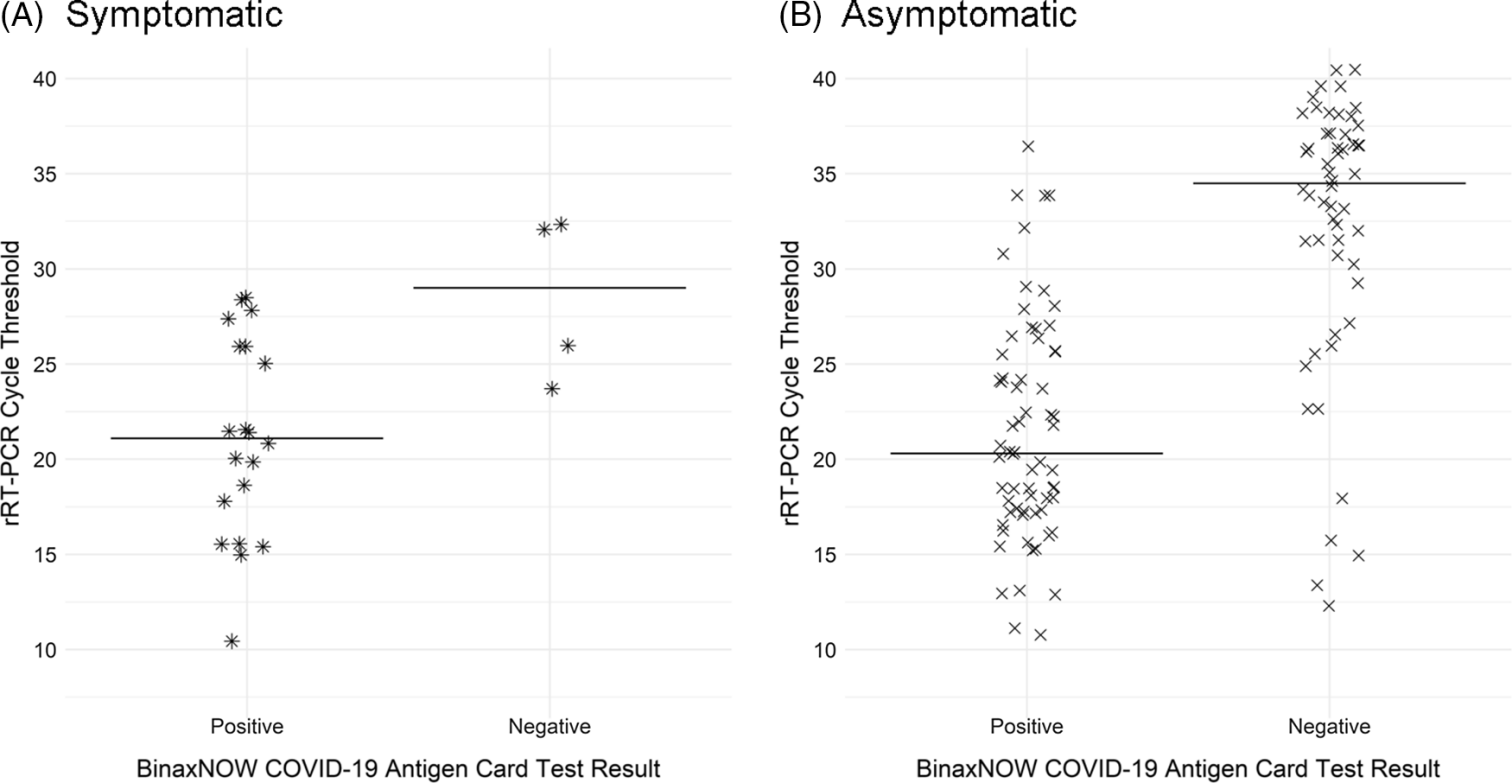
Distribution and median Ct values of SARS-CoV-2 rRT-PCR–positive samples from (A) symptomatic employees and (B) asymptomatic employees by BinaxNOW test result.

Univariate logistic regression analyses of BinaxNOW results relative to Ct values among rRT-PCR positive persons revealed that the odds of a positive antigen test decreased by 20% for each single-cycle increase in rRT-PCR Ct value (OR, 0.8; 95% CI, 0.7–0.9). The odds ratios were similar when stratified by symptom status (Supplementary Table S2 online). Among asymptomatic and symptomatic persons who tested SARS-CoV-2 positive by rRT-PCR, the Ct_mean_ and Ct_median_ were higher among persons who tested negative by BinaxNOW (Fig. 1; Supplemental Table S2 online).

## Discussion

We describe the test performance of the BinaxNOW test relative to the PerkinElmer rRT-PCR assay in the setting of a COVID-19 outbreak among hospital employees. Paired nasal swab testing of 2,339 employees revealed 152 persons who were positive for SARS-CoV-2 by rRT-PCR, 86 (56.6%) of whom were also positive by BinaxNOW. The diagnostic test sensitivity of BinaxNOW was 83.3% among symptomatic employees but only 51.6% in asymptomatic persons. Mean Ct values for persons who tested positive using BinaxNOW and rRT-PCR was lower (Ct_mean_ = 21.4) than for persons who tested BinaxNOW-negative and rRT-PCR–positive (Ct_mean_ = 32.0). This difference was observed among both symptomatic and asymptomatic employees.

Cycle threshold values cannot be used to determine viral load or infectivity in an individual, but on a population level, there is an inverse relationship between Ct value and the amount of genetic material present in specimens.^3^ These analyses demonstrated that asymptomatically infected persons with positive BinaxNOW tests had lower Ct values and, therefore, potentially higher viral loads, which might make them more likely to transmit the virus than asymptomatically infected persons who test negative by BinaxNOW. Despite the low sensitivity of the BinaxNOW test in asymptomatic persons, logistic regression analyses revealed that the sensitivity of the antigen card test improved among rRT-PCR-positive persons as Ct values decreased. Therefore, in an outbreak setting where interrupting transmission quickly is crucial, the benefit of using BinaxNOW could be the ability to rapidly screen and isolate infected persons who may be at greatest risk of transmitting SARS-CoV-2 while awaiting rRT-PCR results.^4^

This study has at least 2 limitations. First, symptom duration was not verified with the participants; thus, we were unable to assess the test performance of BinaxNOW within 7 days of symptom onset. This factor may have lowered the sensitivity of the test compared to the manufacturer’s product information. Second, 29% of eligible employees did not participate in paired BinaxNOW and rRT-PCR testing. Because participants represented a convenience sample, the proportion of persons who were asymptomatic were likely overrepresented relative to the total number of employees because work exclusion and testing protocols were in place for symptomatic persons. Regardless, the impact of this convenience sampling on comparisons between BinaxNOW and rRT-PCR tests was likely minimal.

We report BinaxNOW test parameters relative to the PerkinElmer rRT-PCR assay for SARS-CoV-2 diagnosis during an outbreak of COVID-19 among acute-care hospital employees. The data suggest that, despite the lower sensitivity of the BinaxNOW COVID-19 Ag Card tests, these point-of-care tests could be strategically paired with rRT-PCR testing to immediately identify and isolate persons potentially at higher risk of transmitting the infection while rRT-PCR results are pending.

## Supplementary material

For supplementary material accompanying this paper visit https://doi.org/10.1017/ice.2021.20. click here to view supplementary material
