# Supplementary material for: Performance of the BinaxNOW coronavirus disease 2019 (COVID-19) Antigen Card test relative to the severe acute respiratory coronavirus virus 2 (SARS-CoV-2) real-time reverse transcriptase polymerase chain reaction (rRT-PCR) assay among symptomatic and asymptomatic healthcare employees
Source: Infect Control Hosp Epidemiol. 2021 Jan 25:1–3. doi: 10.1017/ice.2021.20 (PMC7870908; doi:10.1017/ice.2021.20)
Supplement: Supplementary file 1 [file S0899823X21000209sup001.docx]

**Supplementary Material**

**Table of Contents**

**Supplemental Table S1**: Frequencies of symptoms reported (n=115).

**Supplemental Table S2**: Among SARS-CoV-2 rRT-PCR positive samples, frequencies of BinaxNOW test results, mean Ct values, interquartile range of Ct values, and odds ratios of Ct values compared to BinaxNOW test results are reported among all, symptomatic, and asymptomatic employees.

**Supplemental Table S1**: Frequencies of symptoms reported (n=115).

| Symptom Frequency Table | | |
| --- | --- | --- |
| Symptom | Frequency of symptom reported^a^ | Percent^b^ |
| Fever | 7 | 6.1% |
| Cough | 33 | 28.7% |
| Sore throat | 33 | 28.7% |
| Dyspnea | 5 | 4.4% |
| Chills | 7 | 6.1% |
| Headache | 47 | 40.9% |
| Muscle Aches | 14 | 12.2% |
| Vomiting | 0 | 0.0% |
| Abdominal Pain | 5 | 4.4% |
| Diarrhea | 3 | 2.6% |
| Loss of Taste | 0 | 0.0% |
| Loss of Smell | 0 | 0.0% |

^a^Participants could report more than one symptom; frequencies do not sum to 115

^b^Percentage of 115 symptomatic patients reporting symptom

**Supplemental Table S2**. Among SARS-CoV-2 rRT-PCR positive samples, frequencies of BinaxNOW test results, mean Ct values, interquartile range of Ct values, and odds ratios of Ct values compared to BinaxNOW test results are reported among all, symptomatic, and asymptomatic employees.

|  | BinaxNOW Result | N | Ct_mean_ (±SD) | p-value^a^ | Ct_IQR_^b^ | OR (95% CI)^c^ |
| --- | --- | --- | --- | --- | --- | --- |
| All Employees | Negative | 58^d^ | 32.0 (± 7.0) | <.001 | 7.8 | Reference |
|  | Positive | 86 | 21.4 (± 5.7) |  | 8.4 | 0.80 (0.74–0.85) |
|  |  |  |  |  |  |  |
| Asymptomatic Employees | Negative | 66^d^ | 32.3 (± 7.2) | <.001 | 6.9 | Reference |
|  | Positive | 54 | 21.5 (± 5.9) |  | 8.4 | 0.81 (0.75–0.87) |
|  |  |  |  |  |  |  |
| Symptomatic Employees | Negative | 4 | 28.5 (± 4.4) | 0.015 | 7.4 | Reference |
|  | Positive | 20 | 21.1 (± 5.2) |  | 9.3 | 0.69 (0.47–1.0) |

^a^Two-sided Student’s T-test of positive and negative Ct_mean_ within each category (total, asymptomatic, and symptomatic persons)

^b^IQR = Interquartile Range of Ct values

^c^OR=Odds Ratio; CI=Confidence Interval; Univariate logistic regression of individual Ct values compared to BinaxNOW COVID-19 antigen card test result.

^d^Eight asymptomatic employees who received a positive rRT-PCR test were excluded from these analyses because Ct values for the N target were missing; Orf1 target amplification resulted in a positive test for all 8 specimens.
